# ADRD caregiver and care recipient relationship effect on burden, self-efficacy, and perceived social support

**DOI:** 10.1093/geroni/igaf122.1118

**Published:** 2025-12-31

**Authors:** Mariana Stavig, Bailey Gardner, Miriam Jocelyn Rodriguez, Jordan Hill, Lilian Golzarri-Arroyo, Roger Zoh, Richard Holden, Malaz Boustani

**Affiliations:** Indiana University Bloomington, Bloomington, Indiana, United States; Indiana University Bloomington, Bloomington, Indiana, United States; Indiana University Bloomington, Bloomington, Indiana, United States; Indiana University Bloomington, Bloomington, Indiana, United States; Indiana University Bloomington, Bloomington, Indiana, United States; Indiana University Bloomington, Bloomington, Indiana, United States; Indiana University Bloomington, Bloomington, Indiana, United States; Indiana University, Indianapolis, Indiana, United States

## Abstract

**Introduction:**

Relationship types between caregiver and care recipient with Alzheimer’s disease or related dementia (ADRD) effects the caregiving experience. For example, adult child caregivers often juggle caregiving in addition to full-time employment and families of their own, increasing burden. Spouse caregivers report lower levels of social support and increased burden. Caregivers who live with the care recipient report higher rates of burden. The nature of these relationships is important to consider when examining these outcomes.

**Methods:**

Data collected at baseline from participants enrolled in a larger trial (n = 167) were analyzed. Linear regressions examined associations between the caregiver’s relationship to the care recipient and caregiver burden, perceived social support, and self-efficacy. Relationship to the care recipient was categorized into four groups: spouse, child, other family members (e.g., grandchildren, children-in-law), and other (e.g., more complex spousal relationships).

**Results:**

There were no overall significant differences between a caregiver’s relationship and outcome variables. Pairwise comparisons revealed that adult child caregivers and spouses reported marginally lower perceived social support than other family members (p = 0.064 and p = 0.037, respectively). Adult child caregivers had higher rates of self-efficacy in obtaining respite and responding to disruptive behaviors than did spouses (p = 0.032 and 0.014, respectively).

**Discussion:**

Our results identified marginal differences in perceived social support, which align with prior literature and suggest that caregivers in different relational roles experience caregiving in unique ways. Further research is needed to better understand how these dynamics influence caregiving outcomes and to identify targeted interventions that can provide more effective support.

